# Investigating the Integrity of Graphene towards the Electrochemical Hydrogen Evolution Reaction (HER)

**DOI:** 10.1038/s41598-019-52463-4

**Published:** 2019-11-04

**Authors:** Alejandro García-Miranda Ferrari, Dale A. C. Brownson, Craig E. Banks

**Affiliations:** 1 Faculty of Science and Engineering, Manchester Metropolitan University, Chester Street, Manchester, M1 5GD UK; 2 Manchester Fuel Cell Innovation Centre, Manchester Metropolitan University, Chester Street, Manchester, M1 5GD UK

**Keywords:** Graphene, Nanoscale materials, Electrochemistry, Graphene

## Abstract

Mono-, few-, and multilayer graphene is explored towards the electrochemical Hydrogen Evolution Reaction (HER). Careful physicochemical characterisation is undertaken during electrochemical perturbation revealing that the integrity of graphene is structurally compromised. Electrochemical perturbation, in the form of electrochemical potential scanning (linear sweep voltammetry), as induced when exploring the HER using monolayer graphene, creates defects upon the basal plane surface that increases the coverage of edge plane sites/defects resulting in an increase in the electrochemical reversibility of the HER process. This process of improved HER performance occurs up to a threshold, where substantial break-up of the basal sheet occurs, after which the electrochemical response decreases; this is due to the destruction of the sheet integrity and lack of electrical conductive pathways. Importantly, the severity of these changes is structurally dependent on the graphene variant utilised. This work indicates that multilayer graphene has more potential as an electrochemical platform for the HER, rather than that of mono- and few-layer graphene. There is huge potential for this knowledge to be usefully exploited within the energy sector and beyond.

## Introduction

The electrocatalytic splitting of water is considered a promising strategy for the production of hydrogen, which is a clean and carbon neutral fuel with potential applicability in a range of commercial, industrial and transportation sectors^1–3^. The production of molecular hydrogen due to the electrocatalytic splitting of water *via* the Hydrogen Evolution Reaction (HER; 2 H^+^  + 2e^−^ → H_2_) is well-known and improving the overall process, output and costs can give rise to a desirable future source of sustainable energy^4^.

Currently, platinum (Pt) is considered the most active catalyst towards the HER given that it has a small binding energy for the reaction to occur, resulting in the reaction proceeding at low over-potential values close to zero^5–7^. However, Pt is a precious metal with a low natural abundance in the Earth’s crust and has a prohibitive cost for wide-spread implementation in water electrolysers^8^, meaning that for this technology to prevail, an alternative cheaper yet still efficient catalyst is required. As a result of their beneficial properties (in comparison to other, more traditional materials)^9–13^, there is interest in the application of 2D materials, such as graphene^14–17^, graphene oxide^18^, and other (non)carbon nanomaterials^19^ to be explored as Pt alternatives.

For example, Qu *et al*.^20^ have reviewed the use of pristine, doped and hybrid graphene materials for the electrocatalytic splitting of water, indicating that graphene has been subjected to substantial investigation and thus implemented to undertake multiple roles within this area, such as being the electrochemical platform and/or as a functionalizable support^20–29^ for use within a multitude of distinct carbon-based hybrid catalysts. That said, there is a lack of research and understanding with respect to the application of graphene towards the HER in terms of the graphene structure and how the surface changes as a function of electrochemical perturbation.

This paper fully characterises, for the first time, the electrochemical performance of mono-, few- and multilayer graphene electrodes towards the HER, with the purpose of correlating the observed electrochemical behaviour to the change in the physical structure of the graphene surfaces using electrochemical perturbation in the form of electrochemical scanning (linear sweep voltammetry) and Raman analysis.

## Results and Discussion

**Figure 1:**
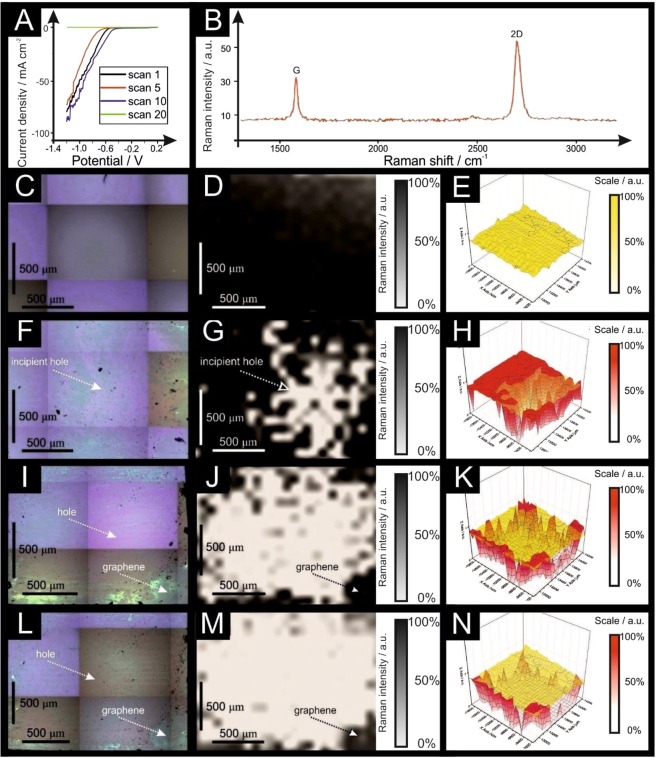
Scanning stability experiments using monolayer graphene; linear sweep voltammetry (LSV) (scan rate: 25 mV s^−1^; *vs*. RHE; solution: 0.5 M H_2_SO_4_). (**B**) Typical Raman profile of the monolayer graphene sheet described and presented in (**C–E**). Optical images of monolayer graphene unused (**C**), after 5 LSV scans (**F**), after 10 LSV scans (**I**) and after 20 LSV scans (**L**). 2D Raman mapping of the monolayer graphene unused (**D**), after 5 LSV scans (**G**), after 10 scans (**J**) and after 20 LSV scans (**M**). 3D Raman mapping of the monolayer graphene sheet unused (**E**), after 5 LSV scans (**H**), after 10 LSV scans (**K**) and after 20 LSV scans (**N**), all in red colour and compared to the unused sheet (yellow overlay). Raman maps show intensity of graphene’s G band (*ca*. 1590 cm^−1^).

Attention was first turned to benchmarking monolayer graphene, without any further modification from the manufacturer, as an electrochemical platform towards the HER within acidic media (0.5 M H_2_SO_4_), as is common within the literature^30,31^; which is the cathode in polymer electrolyte membrane (PEM) electrolysers. A bespoke electrochemistry 3D printed cell was developed and utilised (see Supplementary Information, SI). The physicochemical characterisation of the graphene sample (and others) are presented in the SI, confirming a true monolayer graphene surface. Figure 1A depicts scanning stability experiments using monolayer graphene towards the HER using linear sweep voltammetry (LSV). The initial voltammetric response indicates the HER reaction occurs with an onset value of *ca*. −0.669 V, a current density at −0.65 V of −7.39 mA cm^−2^ and a Tafel slope of 234 mV dec^−1^ (onset and Tafel slope calculated at an overpotential of 10 mA cm^−2^; *vs*. RHE), indicating a reduced electrochemical activity. The HER is less electrochemically reversible than conventional Pt systems (30 mV dec^−1^), which is as expected due to Pt being a pure metal that has a very small binding energy for H^+ ^^5,32^; whereas pristine graphene’s surface is mostly comprised of basal planes, that are reported to have *limited* electrochemical activity^33–36^.

Critically, the effect of electrochemical perturbation, in terms of electrochemical reversibility, stability and cyclability was explored *via* driving the electrochemical potential back and forth multiple times from +0.21 to −1.2 V (*vs*. RHE), the region in which the HER occurs; this type of stability study is often overlooked with the academic literature. Additionally, after each scan, the graphene sample was interrogated with Raman to allow the direct comparison of electrochemical perturbation upon the graphene’s physical structure. Surprisingly, after five successive LSV scans, the current density at −0.65 V was −1.60 mA cm^−2^ with an onset potential of *ca*. −0.859 V and a Tafel slope of 280.5 mV dec^−1^ (*vs*. RHE), showing a decrease in the electrochemical HER performance. Interestingly, after electrochemical perturbation of ten voltammetric scans, an onset value of *ca*. −0.59 V and a current density at −0.65 V of −15.11 mA cm^−2^ is evident, indicating an improvement in the onset potential, however the Tafel slope had changed to 292.0 mV dec^−1^. Furthermore, after a further ten LSV scans (a total of twenty voltammetric scans had been performed), an onset value of *ca*. −0.099 V and a limited current density at −0.85 V of −0.0014 mA cm^−2^ was exhibited, with a Tafel slope of 141.1 mV dec^−1^ describing poor electrochemical activity throughout the HER experiments, indicating an overall significant drop in the electrochemical current and reduced electrochemical activity. These changing electrochemical observations clearly suggest a physical change of the monolayer graphene and in the conductive pathways. In summary, the monolayer graphene initially gives rise to a useful electroactive graphene surface, which upon electrochemical perturbation and potential cycling ultimately reduces to a worse electrochemical response towards the HER.

**Figure 2:**
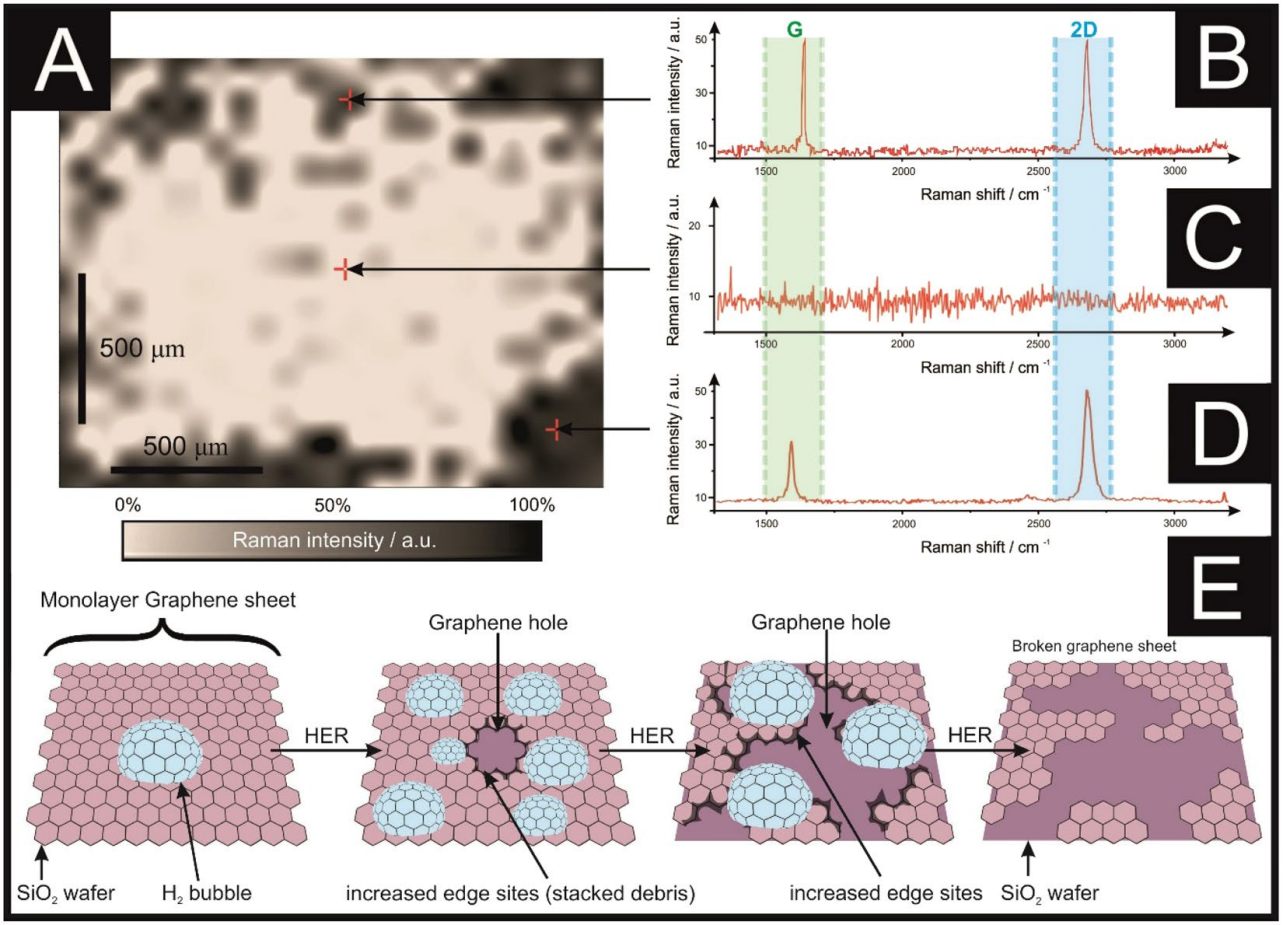
(**A**) Monolayer graphene following 20 LSV scans (Raman map). Part (**B**) depicts the Raman profile near a hole, showing that it is few-layer graphene, with the characteristic ratio of the G/2D peaks near to 1:1. (**C**) Depicts the Raman profile of a broken area where there is no characteristic graphene peak (or signal) present. (**D**) Shows the Raman profile of an intact area where there is monolayer graphene including its typical G (1590 cm^−1^) and 2D (2690 cm^−1^) peaks. (**E**) Is a schematic representation of the behaviour identified within this figure (**A–D**), where the emergence of a bubble on the graphene surface (due to the HER) leads to the creation of some rips when the bubbles move and disperse. The debris created due to the graphene breakdown is stacked in areas near to the holes/rip. When many bubbles explode, there is an incremental rise of the edge sites, caused by the broken graphene pieces, which eventually lead to the complete destruction of the graphene sheet.

In order to understand this phenomenon, Raman mapping of the monolayer graphene surface was explored *after each* LSV scan. Figure 1B to 1N depicts Raman mapping of monolayer graphene following electrochemical perturbation towards the HER, showing how the graphene surfaces changes in-line with the electrochemical data presented in Fig. 1A. Figure 1B represents the typical Raman profile of monolayer graphene. Figure 1C–E depict an unused monolayer graphene surface, indicating that it is a good quality monolayer graphene surface, as demonstrated by the characteristic G and 2D Raman peaks (see Fig. 1B and Supplementary Information for the full characterisation). Five LSV scans were performed and a decrease in the electrochemical performance was observed, followed by mapping with Raman spectroscopy to study the surface of the electrode. Figure 1F–H show the graphene monolayer following five voltammetric scans, where it is evident that the graphene sheet starts to fracture. When more scans are undertaken, it is apparent that one observes the presence of more rips, which correlates to the decreased performance. This phenomena is explored further, with a total of ten voltammetric scans (as depicted in Fig. 1I–K) undertaken. The Raman mapping shows the increased prevalence of holes, with the presence of few and/or multilayer graphene areas (confirmed with Raman spectroscopy as shown in Fig. 2B–D) at the edges of the rips and surrounding the damaged areas. The manifestation of few- and multilayer areas is likely responsible for the improved HER performance (formation of H_2_) reported above. Notably, after twenty LSV scans, there is no electrochemical response and as depicted in Fig. 1L–N, the electrode is completely ripped, such that there is a lack of electrical conductive pathways.

Given the apparent degradation of mono-layer graphene observed above and the presence of few- and multilayer graphene surrounding the damaged areas, total specific capacitance (*C*^0^) calculations are now explored in order to estimate the edge and basal plane coverages of respective ‘pristine’ and ‘damaged’ graphene samples. Total specific capacitance is calculated as described by Eq. 1, which is a weighted average of the edge (*C*_*e*_) and basal plane (*C*_*b*_) contributions comprising the graphene surface^37^:

1 $${C}^{0}={C}_{edge}^{0}\,{f}_{edge}+{C}_{basal}^{0}\,(1-{f}_{edge})$$

where ${C}_{edge}^{0}$ and ${C}_{basal}^{0}$ (in µF cm^−2^) are the specific weighted capacitance averages for edge and basal plane surfaces respectively and ${f}_{edge}$ is the fraction of edge plane on the graphene surface. Previous studies using the *basal plane* of highly ordered pyrolytic graphite (HOPG)^38^ have reported specific capacitance values of *ca*. 1–2 µF cm^−2^, while the specific capacitance of *edge plane* orientated HOPG is *ca*. 70 µF cm^−2^, allowing one to estimate *via* Eq. (1) the relative edge and basal sites of the graphene electrodes utilised herein. Note that previously, the specific capacitance values of graphene (fabricated identically to that used in this study) for 1 to 5 layers have been reported, with values independent to the number of layers but similar in range to that of basal plane HOPG^39^.

Analysis of the edge and basal plane % contribution as a function of electrochemical perturbation (number of scans) is presented in Table 1 (calculated from capacitance tests shown in Fig. S1) which indicates that initially (prior to the first voltammetric scan) the % edge and basal plane is 0.15 and 99.85% respectively. This is in agreement with the voltammetry observed in Fig. 1, where electrochemically irreversible processes are observed due to the low percentage of edge plane coverage and lack of electron transfer sites. Following the fifth LSV scan, the surface changes to 1.24% edge plane and 98.76% basal plane, again correlating with the observed HER performance and Raman mapping experiments. A dramatic change is then observed following ten scans, where the % edge changes to 19.81% and the basal plane to 80.19% respectively, indicating that the graphene surface has a substantial edge plane coverage and thus substantial increase in sites available for fast electron transfer to occur. This agrees with the LSV presented within Fig. 1, where the voltammetric signature becomes more electrochemically reversible. After 20 LSV scans, the % edge plane changes to 8.29% and the % basal plane to 91.71%. Note that adhesively cleaved HOPG has 1–10% edge plane surface coverage^40–42^ suggesting that the mono-layer graphene becomes akin to defect free HOPG. It is important to note that while the surface of the graphene electrode is being damaged, there is also a decrease in the effective area due to graphene detachment from the substrate, which we show in the respective Raman maps as a qualitative measurement. The deduced relative edge and basal plane values support the data observed *via* LSV and Raman mapping, demonstrating how the mono-layer graphene dramatically changes over the course of the HER process, which has not been reported before.

**Table 1:** Determination of the % edge plane sites and % basal plane sites upon the monolayer graphene sheet/electrochemical platform.

Scan Number	Average specific capacitance (µF)	Relative edge plane %	Relative basal plane %
1	0.90	0.15%	99.85%
5	1.86	1.24%	98.76%
10	12.67	19.81%	80.19%
20	6.72	8.29%	91.71%

Cyclic voltammograms were performed within a non-Faradaic region between +0.16 and +0.26 V (*vs*. RHE) at different scan rates (0.1, 0.2 and 0.5 V s^−1^ and analysed to deduce the average specific capacitance and allow the calculation of the edge and basal plane contributions as described by Eq. (1). Solution composition: 0.5 M H_2_SO_4_ (degassed using nitrogen).

A schematic representing the observed changes to the physical surface of monolayer graphene when applied towards the HER is summarised within Fig. 2. As the monolayer graphene starts acting as a catalyst towards the HER, H_2_ bubbles are being generated. Growing bubbles (due to the HER process) can act as collectors of smaller ones, inducing mechanical forces and mass diffusion^43^, that are likely to be responsible of the generation of the first rips on the graphene surface. As shown in Fig. 2A/B, it is evident that the edges of the created holes sometimes fold upon themselves, where the graphene is then present as double layer (as evident in the Raman spectra; note, such spectra is not present prior to electrochemical testing), giving rise to increased edge plane content^44^. Figure 2C shows the Raman profile of the damaged graphene sheet, where there is no presence of the typical G and 2D peaks of the pristine graphene, which usually occur at *ca*. 1590 cm^−1^ and 2690 cm^−1^ respectively^45^, instead only background noise is present due to the SiO_2_/Si wafer onto which the graphene was supported. Given the above insights, the observed initial improvement in the electrochemical performance of monolayer graphene is most likely due to an increase in the planar edge density when a hole is created and the surface is ripped (as depicted schematically in Fig. 2E), after which and with continued ripping the hole becomes too extensive such that there is a loss of the electrical conductive pathways and effectively the graphene sheet ‘disintegrates’ with no further recordable electrochemical signal possible.

The behaviour observed in Figs 1 and 2, and in Table S1 and Fig. S2 depict the evolution of a graphene monolayer during the HER, where holes are created on the electrode surface, likely due to interfacial friction forces caused by H_2_ bubbles growing on top of the graphene, with an estimated size of 4.8 × 10^−7^ mm^3^ to 1.1 × 10^−3^ mm^3^ (supported by Fig. S2 and Table S1 in the Supplementary Information), after which such bubbles burst. The broken graphene debris likely folds onto itself due to Van der Waals forces/attraction, creating a *quasi*^46^ or multilayer graphene area surrounding the edges of the hole (supported with Raman spectroscopy), which leads to an increase in electrochemical behaviour at first (as shown in Fig. 1A). Following that, when the hole is enlarged due to further scanning of the electrode, *via* LSV, the breakdown of the basal layer leads to the creation of more edge plane sites (supported by capacitance tests), hence there is a reduction in the overall electrochemical behaviour or even the full destruction of the graphene monolayer. Note that it has been previously reported that Chemical Vapour Deposition (CVD) grown graphene and SiO_2_ substrates have different wettability properties, although underlying substrates do not seem to affect the wettability of such graphene^47^; in the presence of graphene with rips and holes as this manuscript reports, is likely that the different areas might have different wettability, such effect needs further exploration and will be investigated in the future.

In order to gain further insights, it is interesting to compare the above results with few- and multilayered graphene, applying the same analysis of stability and cyclability experiments towards the HER. Thus, we next investigate if the thickness of graphene layers has an impact on the structural integrity.

**Figure 3:**
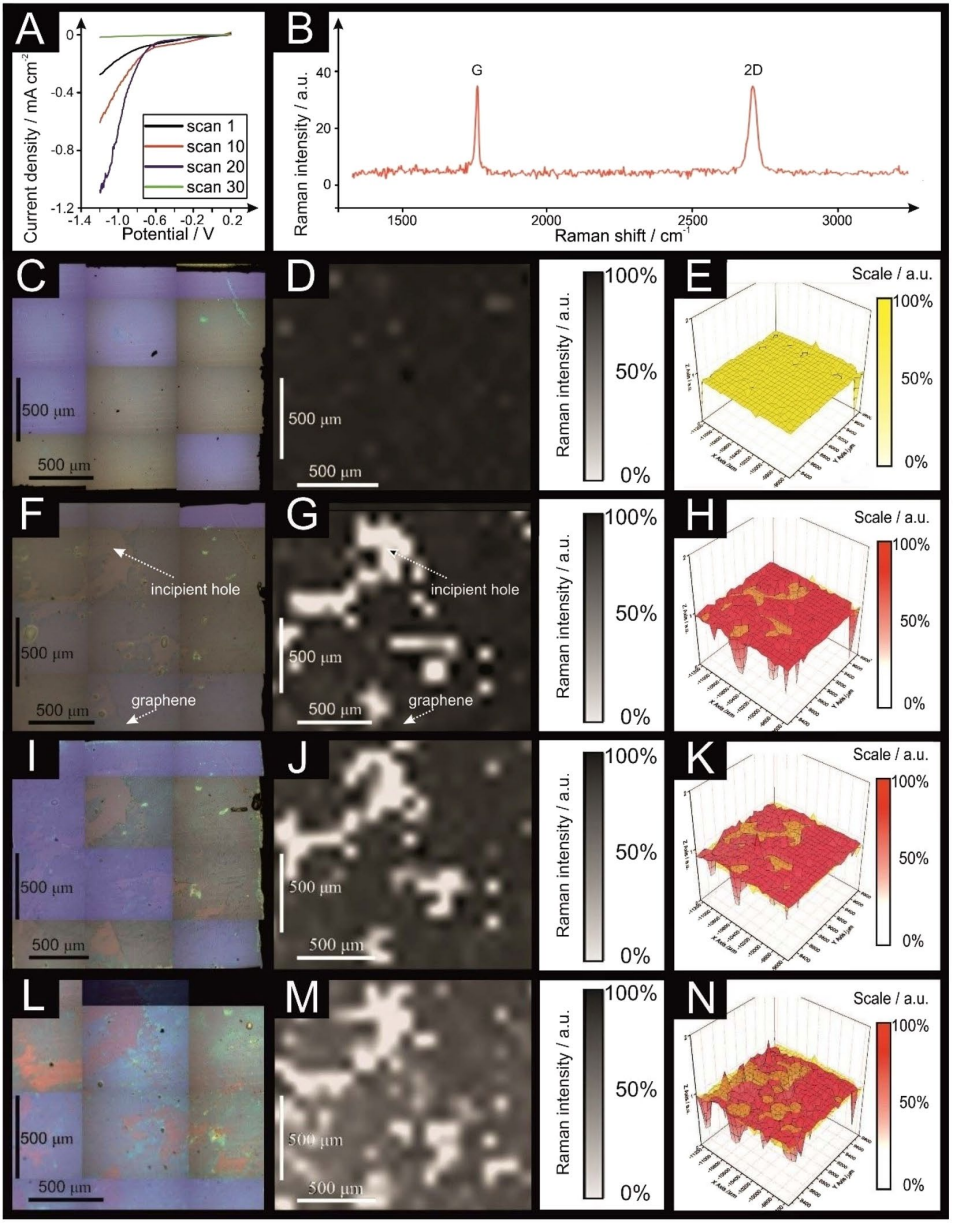
Scanning stability experiments using few-layer graphene; linear sweep voltammetry (LSV) (**A**; scan rate: 25 mV s^−1^; *vs*. RHE; solution: 0.5 M H_2_SO_4_). (**B**) Typical Raman profile of the few-layer graphene described and presented in (**C–E**). Optical images of a few-layer graphene unused (**C**), after 10 LSV scans (**F**), after 20 LSV scans (**I**) and after 30 LSV scans (**L**). 2D Raman mapping of the few-layer graphene unused (**D**), after 10 LSV scans (**G**), after 20 LSV scans (**J**) and after 30 LSV scans (**M**). 3D Raman mapping of the few-layer graphene unused (**E**), after 10 LSV scans (**H**), after 20 LSV scans (**K**) and after 30 LSV scans (**N**), all in red colour and compared to the unused sheet (yellow overlay). Raman maps show intensity of graphene’s G band (*ca*. 1590 cm^−1^).

**Figure 4:**
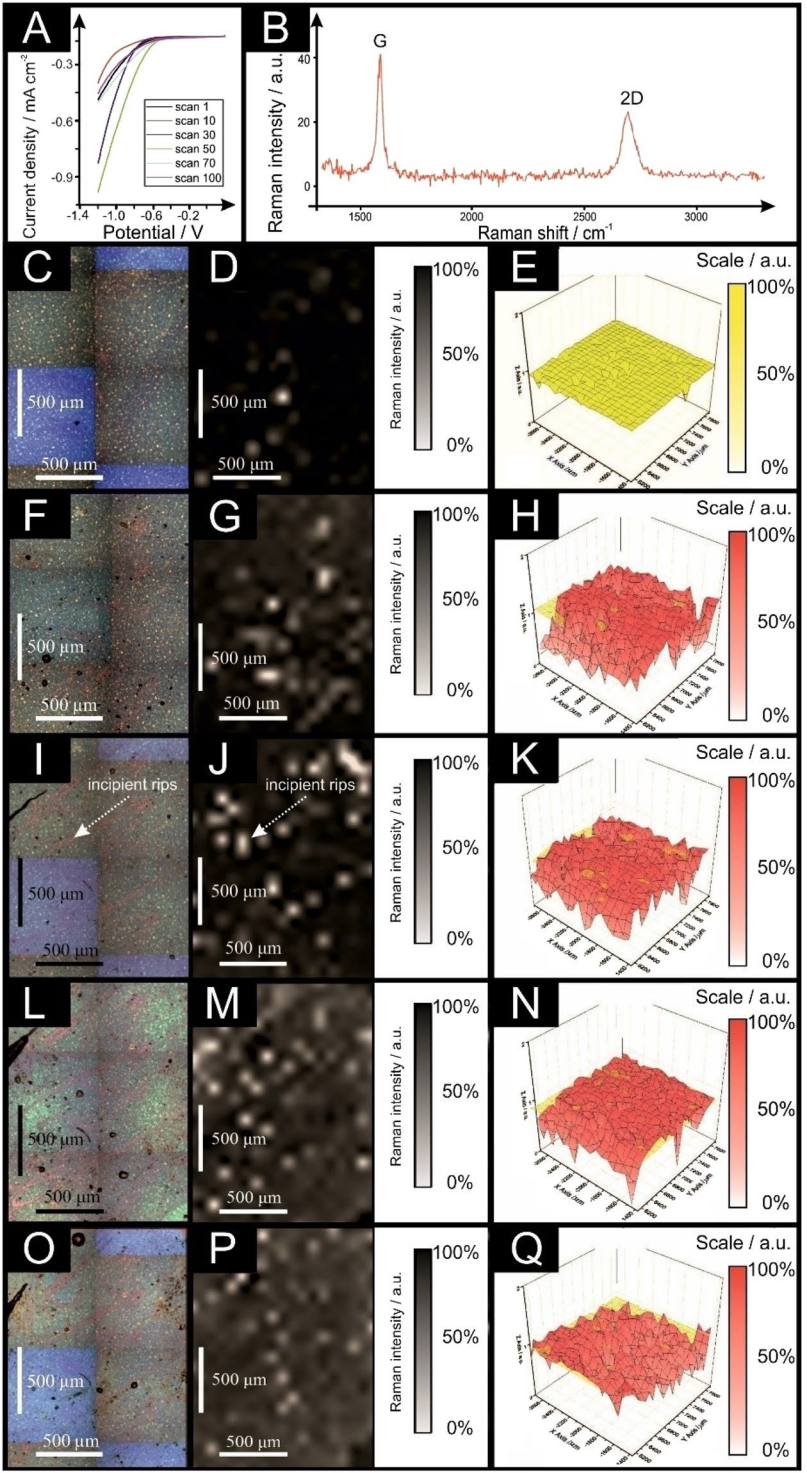
Scanning stability experiments using multilayer graphene; linear sweep voltammetry (LSV) (**A**; scan rate: 25 mV s^−1^; *vs*. RHE; solution: 0.5 M H_2_SO_4_). (**B**) Typical Raman profile of the multilayer graphene described and presented in (**C–E**). Optical images of a multilayer graphene unused (**C**), after 30 LSV scans (**F**), after 50 LSV scans (**I**), after 70 LSV scans (**L**) and after 100 LSV scans (**O**). 2D Raman mapping of the multilayer graphene unused (**D**), after 30 LSV scans (**G**), after 50 LSV scans (**J**), after 70 LSV scans (**M**) and after 100 LSV scans (**P**). 3D Raman mapping of the multilayer graphene unused (**E**), after 30 LSV scans (**H**), after 50 LSV scans (**K**), after 70 LSV scans (**N**) and after 100 LSV scans (**Q**), all in red colour and compared to the unused sheet (yellow overlay). Raman maps show intensity of graphene’s G band (*ca*. 1590 cm^−1^).

Few- and multilayer graphene electrodes were explored towards the HER (as shown in Figs 3A and 4A respectively). Few-layer graphene was tested for a maximum of thirty voltammetric scans, showing a change in the current density (at −0.65 V) from −0.065 mA cm^−2^ to −0.0042 mA cm^−2^. In contrast, the multilayer graphene sheet increased its current density from −0.028 mA cm^−2^ to −0.41 mA cm^−2^ when scanned *one hundred times*. Evidently, the surface of few-layer graphene is susceptible to similar surface changes as observed with monolayer graphene (but to a less significant degree than the latter), whereas multilayered graphene remains unaltered and stable throughout experiments (with an intrinsic resistance to such surface changes apparent).

We now turn to analysing the surface of the few- and multilayer graphene electrodes with Raman mapping spectroscopy. In order to study how the number of layers affects the integrity of the graphene sheet when utilised in electrochemistry, as depicted in Figs 3 and 4 respectively.

The few-layer graphene electrode is confirmed to be a continuous good quality sheet *via* the Raman and optical images that are presented. Electrochemical scanning of the HER potential window ten times evidently creates holes in the sheet, as shown in Fig. 3F–H. Following an additional twenty scans, a major damaged area is evident on the electrode surface, which is confirmed with Raman spectroscopy in Fig. 3L–N; however, one must be aware that the disruption to the electrode surface is not to the same extent as that observed previously with monolayer graphene as it is likely that underlying layers dissipate the effect of lost upper layers and hence a loss in electrical conductivity.

Finally, multilayer graphene was analysed *via* Raman mapping and shown to be approaching the structural configuration of graphite (see Fig. 4B), as confirmed in Fig. 4C–E. Note that although minor changes to the electrode surface are observable following fifty LSV scans of the HER potential window, this remains unchanged up to one hundred scans (Fig. 4F–Q). Clearly however, the Raman maps remain unaltered and there is little change in the HER performance observed, such that with the multilayered graphene electrode there appears to be an inherent protection against the breakdown of the graphitic structure. The origin of this maintained integrity is likely that of the underlying graphene layers (when exposed due to the failure of the upper most layers) reacting identically to the first layer and thus maintaining the observed response.

## Conclusions

In summary, this paper indicates that mono- and few-layer graphene, when used as electrodes toward the HER, break-up when an electrical current is applied to them during electrochemical HER experiments performed within aqueous solution. The mechanism of which is first ripping of the film due to the evolution of H_2_ (*i.e*. bubbles), creating surface defects due to cavitational forces and a large edge plane % (*i.e*. causing an observed improved response), after which ultimately the integrity of the graphene film as a whole is not viable and the electrical conductive pathways are disrupted and result in a loss of electrochemical signal. This response is mirrored with few-layered graphene structures, but not to the same extent. Conversely, multilayered graphene structures do not present this phenomena and remain stable (HER values and film integrity) after HER scanning for an extensive number of scans. These findings are of high importance to those working in the graphene energy field, particularly for those designing and implementing graphene electrical components given that the sheet integrity is questioned herein and this report shows that pristine graphene is not a beneficial electrode material towards the HER.

### Experimental section

All chemicals used were analytical grade and were used as received from the supplier (Sigma-Aldrich, Irvine, UK) without any further purification. All solutions were prepared with deionised water of resistivity no less than 18.2 MΩ cm and were vigorously degassed prior to electrochemical measurements with high purity, oxygen free nitrogen. The tested solutions were 0.5 M H_2_SO_4_.

Electrochemical measurements were performed using a three-electrode system on an Autolab PGSTAT204 potentiostat (Metrohm Autolab, Utrecht, The Netherlands). Working electrodes were: commercially obtained chemical vapour deposition (CVD) synthesised mono-layer, a few-layer (*quasi*-graphene) and multilayer-graphene films supported on an oxidised silicon wafer. A Pt wire counter/auxiliary electrode and a silver/silver chloride (saturated Ag/AgCl; +0.210 V *vs*. RHE) reference electrode completed the circuit.

The commercially available CVD synthesised graphene films, that have been used in our previous work^44,48,49^, were obtained from ‘Graphene Supermarket’ (Reading, MA, USA)^50^ and are known as ‘graphene on 285 nm SiO_2_ Wafer’ and have been previously reported and characterised in the literature^48,51–53^; the exact details are proprietary information^50^. Note that full physicochemical characterisation (Raman spectroscopy and X-ray photoelectron spectroscopy (XPS)) of the various graphene samples utilised within this work is reported in the Supplementary Information.

The graphene working electrode was secured into a bespoke 3D printed electrochemical cell that is described fully in the Supplementary Information (Fig. S4) and is connected with copper foil to a crocodile-clip connector that allows electrical connection to the potentiostat and external reference and counter electrodes. SolidWorks software was used to design the 3D printed electrochemical cell, which was been printed using a UV curable proprietary polymer and a ‘Form 2’ 3D printer from Formlabs (Somerville, MA, USA).

Raman Mapping Spectroscopy data was performed using a DXR Raman Microscope (Thermo Scientific, UK) fitted with a 532 nm excitation laser at a low power of 3 mW to avoid any heating effects. Spectra were recorded using a 3 seconds exposure time for 3 accumulations at each point. To collect the map a step size of 75 × 75 µm and a Raman profile between the region of 1050 and 3300 cm^−1^ was employed. Raman maps show intensity of graphene’s G band (*ca*. 1590 cm^−1^, which is related to the first order Raman band for all sp^2^ hybrised carbons) in order to show the presence of graphene. It is important to note that it has not been detected the presence of D or D’ Raman band in the samples, which is likely to be due to the large sample size (centimetre scale) with newly-created defects in the macroscale. In this case, it is only detected the presence of graphene (in different number of layers) or the lack of graphene presence (lack of graphene Raman peaks with only SiO_2_ Raman peaks present).

The Tafel analysis method is utilised in the academic literature to describe the underlying electrochemical processes of the HER, and are an indicator of the reaction mechanism. The Volmer-Heyrovsky-Tafel mechanism includes^27^:

the initial H^+^ discharge step being the Volmer reaction, leading to the following equation^3,28,29^:

2 $${H}_{3}{O}^{+}+(aq)+{e}^{-}+catalyst\to H\,(ads)+{H}_{2}O\,(l)$$

$$\frac{2.303RT}{\propto F}\approx 120\,mV$$

the Volmer step can then followed by one of two possible steps; either the Heyrovsky step:

3 $$H\,(ads)+{H}_{3}{O}^{+}(aq)+{e}^{-}\to {H}_{2}\,(g)+{H}_{2}O\,(l)$$

$$\frac{2.303RT}{(1+2)F}\approx 40\,mV$$

or the Tafel step:

4 $$H\,(ads)+H\,(ads)\to {H}_{2}\,(g)$$

$$\frac{2.303RT}{(2)F}\approx 30\,mV$$

where the transfer coefficient (∝) is assumed to be 0.5, *F* is the Faraday constant, *R* is the universal gas constant and *T* is the temperature at which the electrochemical experiment was performed, each step is capable of being the rate-determining step of the HER. This analysis is dependent on the corresponding Tafel slope.

The Tafel Slope and onset values have been calculated at 10 mA cm^−2^ from the plot of log current density *vs*. potential values.

## Supplementary information

Supplementary information
