# Supplementary material for: Investigating the Integrity of Graphene towards the Electrochemical Hydrogen Evolution Reaction (HER)
Source: Sci Rep. 2019 Nov 4;9:15961. doi: 10.1038/s41598-019-52463-4 (PMC6828781; doi:10.1038/s41598-019-52463-4)
Supplement: Supplementary file 1 — Supplementary information [file 41598_2019_52463_MOESM1_ESM.pdf]

Supplementary Information:

Investigating the Integrity of Graphene towards the Electrochemical Hydrogen Evolution Reaction (HER)

Alejandro García-Miranda Ferrari[a,b], Dale A. C. Brownson[a,b], and Craig E. Banks[a,b]*

[a]: *Faculty of Science and Engineering, Manchester Metropolitan University, Chester Street, Manchester M1 5GD, UK.*

[b]: *Manchester Fuel Cell Innovation Centre, Manchester Metropolitan University, Chester Street, Manchester M1 5GD, UK*

Table S1. Hydrogen bubble size growth generated *in-situ* while performing chronoamperometry, potential held at -1.2 V *vs*. RHE, using monolayer graphene, which clearly shows the evolution of a single hydrogen bubble from its initial generation to its explosion. Note that the measurement of the bubble started once it was big enough to be analysed with the optical microscope, therefore we timed that initial measurement as 'time 0 seconds'.

Time (s)	*Diameter of bubble (μm)*	*Volume of bubble (mm^3)*
0.0	9.7	4.8×10^{-7}
1.0	36.4	2.5×10^{-5}
3.0	54.9	8.7×10^{-5}
5.0	77.1	2.4×10^{-4}
20.0	151.8	1.8×10^{-3}
45.0	219.9	5.6×10^{-3}
72.5	272.8	1.1×10^{-2}

Figure S1. Voltammograms of a non-faradaic region between +0.16 and +0.26 V analysed to calculate the data shown in Table S1. In all cases the scan rate was 100 mVs^{-1} (*vs*. RHE) and the solution composition was 0.5 M H_2SO_4 (degassed using nitrogen).

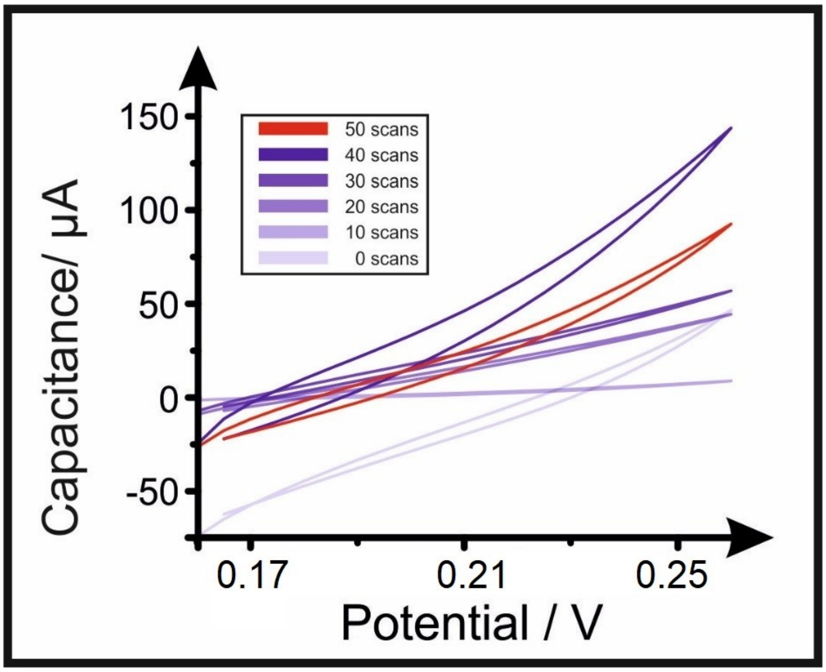

Figure S2. Snapshot from an *in-situ* video recorded while performing chronoamperometry, (potential held a -1.2 V (*vs*. RHE)) using monolayer graphene clearly showing the evolution of hydrogen bubbles on top of the graphene electrode over the following time periods: 0 (A) (zero as initial measurement time), 1 (B), 4 (C), 14 (D), 45 (E) and 67 (F) seconds. Video recorded from the top of the graphene during the electrochemical experiment.

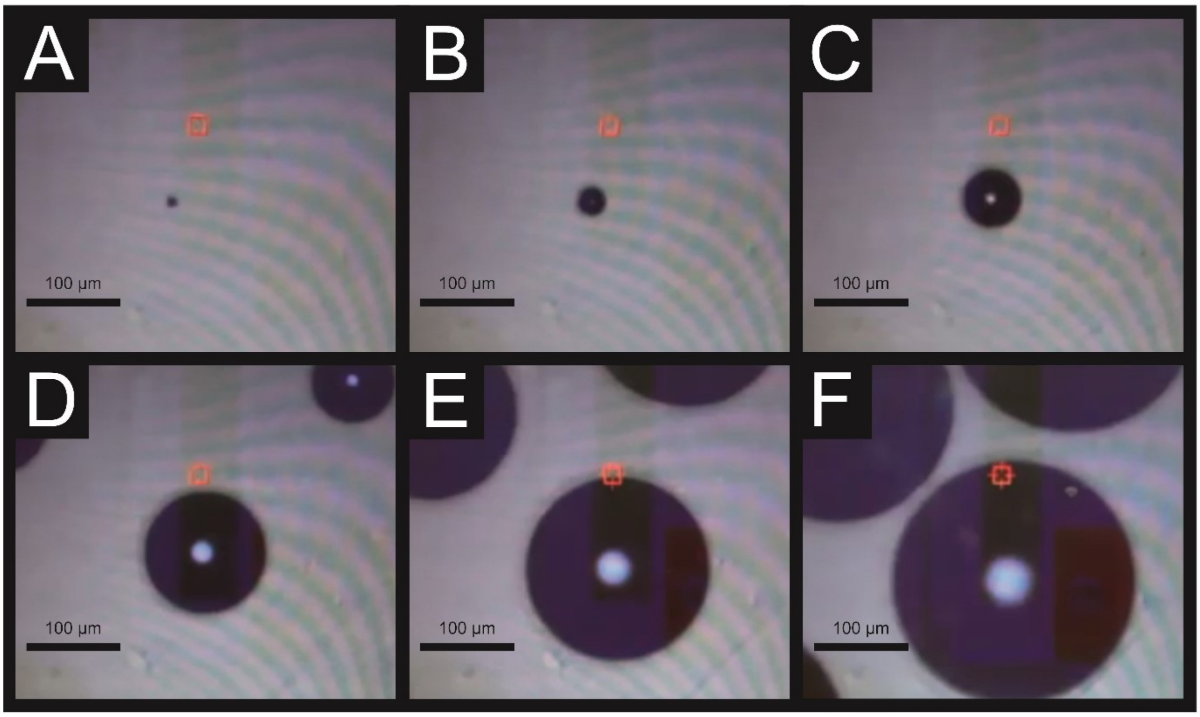

Physicochemical characterisation of the graphene used in this work

The physicochemical characterisation of the batch graphene samples used in this work and their characterisation is reported below.

Atomic force microscopy (AFM) characterisation of the batch graphene samples used in this work including the monolayer and multilayer graphene have been reported previously in Ref [2]. Furthermore, X-ray photoelectron spectroscopy (XPS) has previously been performed on these batch samples revealing the monolayer graphene to comprise of an O/C ratio of *ca*. 0.05, which is consistent with that of a low oxygen content of the graphene domain and thus is indicative of being pristine (*aka* pristine graphene).[1] In the case of the multilayer graphene samples, XPS reveals a O/C ratio of *ca*. 0.07, that is again consistent with inferences gained through Raman spectroscopy (see later) and indicates that the this material is comprised of pristine graphene.

Raman characterisation of the batch mono-, few- and multilayer graphene electrode normalised to the G peak were performed and are as depicted in Figure S3. The Raman spectra of the graphene films confirms the G (*ca*. 1550 cm^{-1}) and 2D (*ca*. 2680 cm^{-1}) characteristic peaks that allow us to quantify the number of graphene layers. The Raman spectra of the monolayer graphene sheets reveals that the full width at half-maximum (FWHM) of the 2D band corresponds to 34.72 cm^{-1}, which upon exploring the literature[3-4] indicates that our batch samples are comprised of single layer of graphene; additionally the intensity ratio G/2D of 0.72 suggests that the graphene samples are comprised of monolayer due to the lower intensity of the G band in relation to the 2D peak. The Raman spectrum of the few-layer graphene films reveals an intensity ratio of G/2D of 1.00 suggesting that the electrode is comprised of dual-layer graphene, but as there are occasional multilayer islands; it is therefore named herein as “few-layer” graphene. The Raman spectrum of the multilayer graphene reveals an intensity ratio of G/2D of 1.76 suggesting that such electrode is comprised of multilayer graphene.

Figure S3. Raman characterisation of mono-, few- and multilayer graphene utilised within this work. Raman is performed with a 532 nm excitation laser at a low power of 3 mW to avoid any heating effects. Spectra were recorded using a 3 seconds exposure time for 3 accumulations at each point.

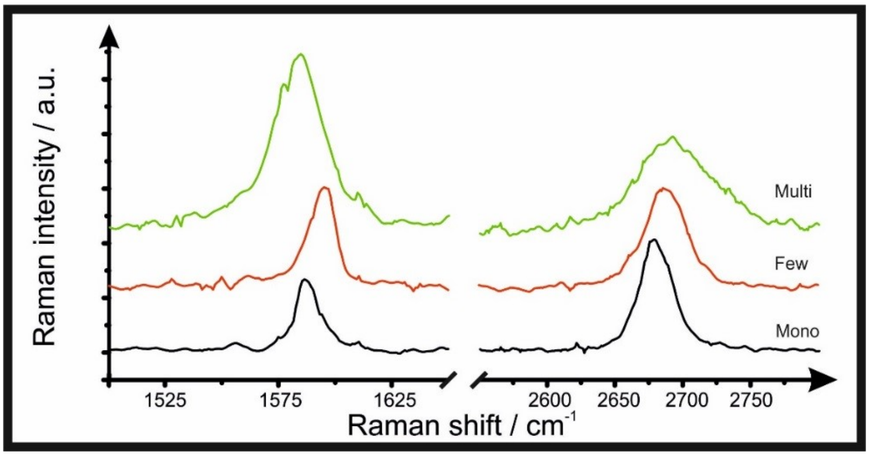

Graphene housing 3D printed cell

An image of the graphene 'housing' 3D printed cell (A) designed using Solidworks and printed using a Form 2 3D UV curable printer. Perspective (B) and cross-sectional (C) view of the assembled CVD graphene chip when reference and counter electrodes are incorporated into the three electrode system in the 3D printed cell. Schematic diagram of the graphene 'housing' 3D printed cell (D). The cell allows the connection of the graphene chip to the potentiostat with a copper foil wrapping the graphene chip and allowing its use in a microscope or Raman Spectrometer without dismounting and manipulating the chip. There is a silicon O-ring sealing the graphene chip, keeping constant the studied area of the working electrode (WE). There is extra space to contain the liquid solution and the external reference (RE) and counter electrodes (CE).

Figure S4. Schematic of graphene housing 3D printed cell, including a photograph of the mounted cell (A), 3 schematics from different perspectives (B, C and D) and a zoomed in perspective of the graphene WE showing how the copper connects the graphene to the potentiostat and how the use of the O-ring prevents any leaks. A Pt wire counter/auxiliary electrode and a silver/silver chloride (saturated Ag/AgCl; +0.210V *vs.* RHE) reference electrode completed the circuit. This schematic is part of previous work from our research group[5].

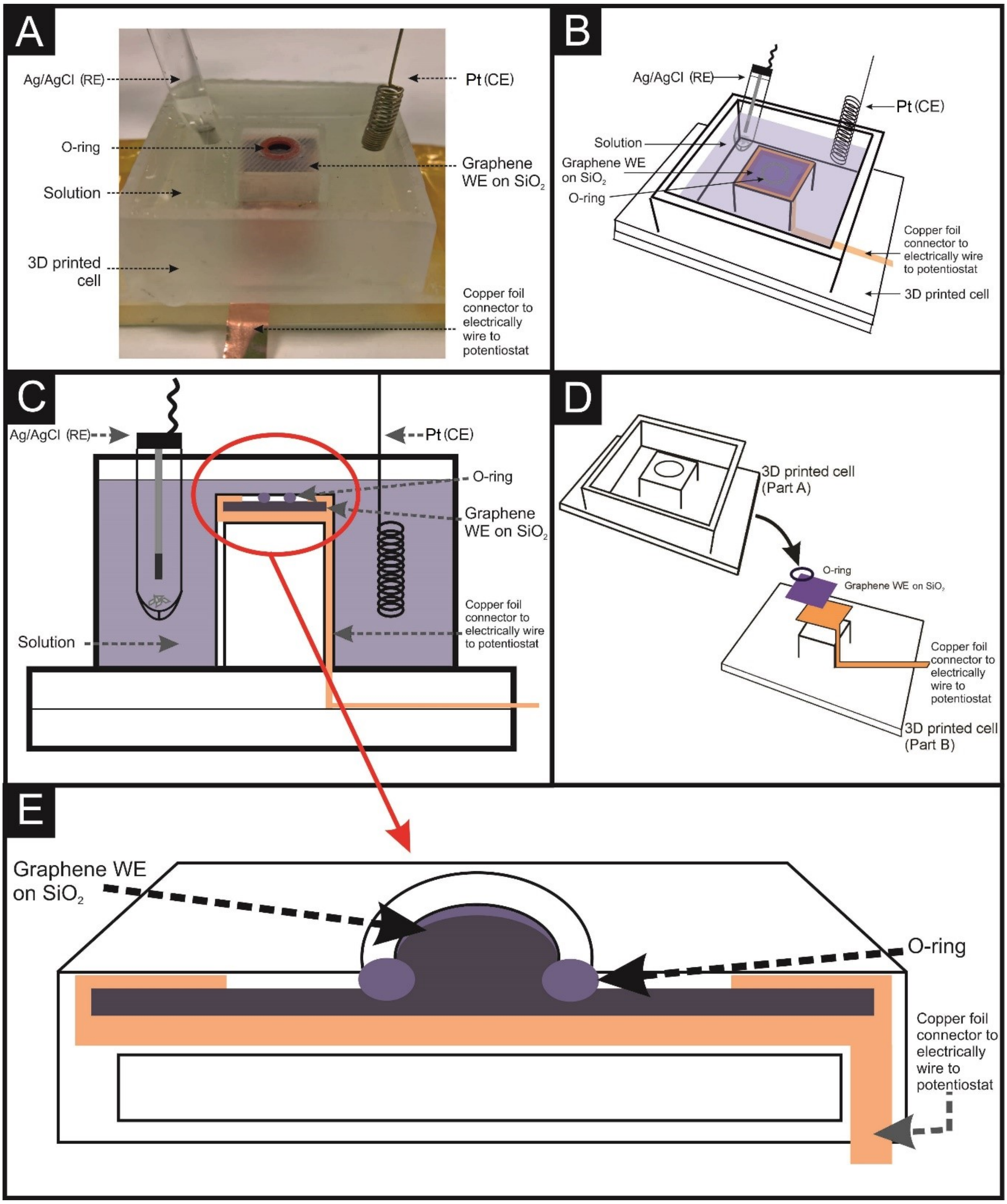

References

1. Smith, R. E. G., Davies, T. J., Baynes, N. D. and R. Nichols, J.. The electrochemical characterisation of graphite felts. *J Electroanal Chem* **747**, 29-38 (2015).
2. Brownson, D.A.C., Varey, S. A., Hussain, F., Haigh, S. J. and Banks, C.E.. Electrochemical properties of CVD grown pristine graphene: monolayer- vs. quasi-graphene. *Nanoscale* **6**, 1607-1621 (2014).
3. Ferrari, A. Raman spectroscopy of graphene and graphite: Disorder, electron–phonon coupling, doping and nonadiabatic effects. *Solid State Commun* **143**(1), 47-57 (2007).
4. Graf, D., et al.. Spatially Resolved Raman Spectroscopy of Single- and Few-Layer Graphene. *Nano Lett* **7** (2), 238-242 (2007).
5. Garcia-Miranda Ferrari, A., Foster, C.W., Brownson, D.A.C., Whitehead, K.A. and Banks, C.E.. Exploring the reactivity of distinct electron transfer sites at CVD grown monolayer graphene through the selective electrodeposition of MoO_2 nanowires. *Sci Reps* **9** (1) (2019).
